# External Validation of the RESCUE-IHCA Score as a Predictor for In-Hospital Cardiac Arrest Patients Receiving Extracorporeal Cardiopulmonary Resuscitation

**DOI:** 10.5811/westjem.18601

**Published:** 2024-10-04

**Authors:** Yi-Ju Ho, Pei-I Su, Chien-Yu Chi, Min-Shan Tsai, Yih-Sharng Chen, Chien-Hua Huang

**Affiliations:** * National Taiwan University Hospital, Department of Emergency Medicine, Taipei, Taiwan; † National Taiwan University Hospital Yun-Lin Branch, Department of Emergency Medicine, Yun-Lin, Taiwan; ‡ National Taiwan University Hospital, Department of Internal Medicine, Taipei, Taiwan; § National Taiwan University Hospital, Department of Surgery, Taipei, Taiwan; ∥ National Taiwan University, College of Medicine, Department of Surgery, Taipei, Taiwan

## Abstract

**Background:**

Extracorporeal cardiopulmonary resuscitation (ECPR) improves the prognosis of in-hospital cardiac arrest (IHCA). The six-factor RESCUE-IHCA score (resuscitation using ECPR during IHCA) was developed to predict outcomes of post-IHCA ECPR-treated adult patients. Our goal was to validate the score in an Asian medical center with a high volume and experience of ECPR performance and to compare the differences in patient characteristics between the current study and the original cohort in a 2022 observational study.

**Method:**

For this single-center, retrospective cohort study we enrolled 324 ECPR-treated adult IHCA patients. The primary outcome was in-hospital mortality. We used the area under the receiver operating curve (AUROC) to externally validate the RESCUE-IHCA score. The calibration of the model was tested by the decile calibration plot as well as Hosmer–Lemeshow goodness-of-fit with an associated *P*-value.

**Results:**

Of the 324 participants, 231 (71%) died before hospital discharge. The discriminative performance of the RESCUE-IHCA score was comparable with the originally validated cohort, with an AUC of 0.63. A prolonged duration of cardiac arrest was associated with an increased risk of mortality (odds ratio [OR] 1.02, 95% confidence interval [CI] 1.01–1.03, *P* = .006). An initial rhythm of ventricular tachycardia (OR 0.14, 95% CI 0.04–0.51, *P* = .003), ventricular fibrillation (OR 0.11, 95% CI 0.03–0.46, *P* = .003), and palpable pulse (OR 0.26, 95% CI 0.07–0.92, *P* = 0.04) were associated with a reduced mortality risk compared to asystole or pulseless electrical activity. In contrast to the original study, age (*P* = 0.28), resuscitation timing (*P* = 0.14), disease category (*P* = 0.18), and pre-existing renal insufficiency (*P* = 0.12) were not associated with in-hospital death.

**Conclusion:**

In external validation, the RESCUE-IHCA score exhibited performance comparable to its original validation within the single-center population. Further investigation on hospital experience, time-of-day effect, and specific disease categories is warranted to improve the selection criteria for ECPR candidates during IHCA.

Population Health Research Capsule What do we already know about this issue?

*Extracorporeal cardiopulmonary resuscitation improves the prognosis of in-hospital cardiac arrest. The RESCUE-IHCA score predicts outcomes for these patients.*

What was the research question?

*We aimed to validate the RESCUE-IHCA score and to compare differences in patient characteristics between our study and a 2010 observational study.*

What was the major finding of the study?

*The RESCUE-IHCA score showed compromised discrimination compared to the original study, with an AUC of 0.63 (95% CI 0.56–0.70).*

How does this improve population health?

*The RESCUE-IHCA score did not predict outcomes better than the originally validated cohort. Method of disease categorization may have influenced outcomes.*

## INTRODUCTION

Extracorporeal cardiopulmonary resuscitation (ECPR) is a promising modality that combines extracorporeal membrane oxygenation (ECMO) with traditional CPR techniques for improving the outcome after cardiac arrest. Prediction models developed to estimate the survival likelihood of patients with refractory cardiogenic shock or cardiac arrest who received ECPR have rarely focused on patients who have sustained an in-hospital cardiac arrest (IHCA),^1^ ^,^ ^2^ A 2010 observational study showed that ECPR leads to more favorable outcomes in IHCA than in out-of-hospital cardiac arrest (OHCA),^3^ possibly owing to the shorter no-flow and low-flow duration. Other observational studies have attempted to ascertain strong predictors that can help identify IHCA patients who would benefit most from ECPR.^4^ ^,^ ^5^

The RESCUE-IHCA scoring system (resuscitation using ECPR during IHCA) was developed to predict outcomes of ECPR-treated adult IHCA patients and was externally validated using patient data from the Extracorporeal Life Support Organization (ELSO) Registry.^6^ RESCUE-IHCA is a simplified score that comprises six variables: 1) age; 2) pre-existing renal insufficiency; 3) time of the day (7 am – 2:59 pm); 4) disease category (cardiac, or non-cardiac, surgical or medical, as per the Current Procedural Terminology and International Classification of Diseases); 5) initial rhythm; and 6) the duration of arrest, all of which can be easily collected upon hospital arrival. However, in the validation group, the RESCUE-IHCA scoring system only demonstrated acceptable discrimination.

Despite its modest clinical performance, the RESCUE-IHCA is the only model available for predicting outcomes of ECPR-treated IHCA patients. Therefore, further evaluation of the RESCUE-IHCA’s reproducibility by using external datasets is warranted for wider application of this scoring system. Our objective was to validate the RESCUE-IHCA score using data from a different population and to identify potential predictors that may differ from those in the original study. We aimed to enhance clinical decision-making by providing more accurate outcome predictions for ECPR initiation in IHCA patients.

## METHODS

### Study Design and Setting

This retrospective cohort study was conducted over a seven-year period (January 2012–December 2019) at a tertiary, extracorporeal life-support referral medical center in Taiwan, one of the largest medical centers in Asia with 2,600 beds, including 220 beds in intensive care units. Most patients are Taiwanese residents, with foreigners occasionally admitted through international transfer. Over the past decade, the medical center has performed more than 100 ECPR procedures annually under the guidance of cardiac surgeons.^7^ This study, approved by the institutional review board [202306052RIND], demonstrates a robust adherence to methodological standards in health record review studies.^8^ The requirement of informed consent was waived due to the retrospective nature of the research. The sampling patients were identified through chart review of electronic health records (EHR) by medical and emergency physicians who collected covariate data and defined the post-IHCA outcome.

### Case Selection

Between January 2012–December 2019, study participants were enrolled based on these selection criteria: 1) patients had undergone ECPR following IHCA; 2) were aged ≥18 years; and (3) had no history of OHCA prior to admission. We used a critical screening process to exclude ineligible patients based on the following criteria: 1) transfer to another hospital after return of spontaneous circulation; 2) traumatic arrest; 3) history of OHCA; and 4) missing outcomes in the EHR.

### Data Collection and Processing

Covariate data from each medical chart were defined clearly and reviewed by independent physicians, and monthly meetings were held to ensure consistency of the collected data. To minimize potential biases or errors, the study design and data analysis were undertaken by a physician who was blinded to the data collection process. We discussed any disputes or ambiguous records with cardiologists and emergency physicians, and decisions were made regarding each controversial health record. Individuals who lacked outcome variables were excluded initially. The only missing data in the current cohorts was the pre-arrest laboratory data, which was not included in the RESCUE-IHCA score or the final analysis.

### Variables

We categorized the study variables into demographics, pre-existing diseases, intra-arrest characteristics, and presumed etiology of cardiac arrest. Demographics included age, gender, body weight, and body mass index (BMI). Pre-existing diseases included hypertension, diabetes mellitus, chronic obstructive pulmonary disease, coronary artery disease, congestive heart failure, chronic kidney disease, cerebral vascular disease, and cancer, and the diagnoses were confirmed from the EHR based on regular medication prescriptions, treatment, and outpatient follow-up. Intra-arrest characteristics included initial cardiac rhythm defined as asystole, pulseless electrical activity, ventricular tachycardia (VT), ventricular fibrillation (VF), or with a palpable pulse initially; time of day, duration of cardiac arrest; and intra-arrest treatments including defibrillation and medications administered. The presumed etiology of IHCA was determined by reviewing the EHR. We categorized participants into four groups based on whether the IHCA was cardiogenic or non-cardiogenic and was related to a surgical or medical illness (Supplementary Table 1).

### Outcomes

As with the outcome of the original RESCUE-IHCA study, the primary outcome in this study was in-hospital death. We calculated the RESCUE-IHCA score in our datasets for the external validation process.

### Statistical Analysis

Continuous data were assessed for normality using the Kolmogorov–Smirnov test and were expressed as the mean (standard deviation) if normally distributed or median (interquartile range [IQR]) if non-normally distributed. We presented dichotomous and categorical variables as the frequency (percentage). We compared continuous variables using the Mann-Whitney *U* test, whereas dichotomous and categorical variables were examined using the chi-square test. We used the Hosmer-Lemeshow test to show the goodness of fit.

The external validation of the RESCUE-IHCA score was performed in the study cohort, and we assessed discriminatory performance using the area under the receiver operating characteristic curve (AUROC) with 95% confidence interval (CI). The model calibration was tested using a calibration plot based on 10 deciles, as well as the Hosmer–Lemeshow goodness-of-fit test with an associated *P*-value. We tested individual variables with a binary logistic regression model and adjusted them in the multivariate regression model using the force-entry method. The results were presented as adjusted odds ratio and 95% CI.

We performed statistical analysis using the Statistical Package for the Social Sciences version 26.0 (IBM Corp, Armonk, NY) and R 4.3.0 (R Foundation for Statistical Computing, Vienna, Austria). A two-sided *P*-value less than 0.05 was considered statistically significant.

## RESULTS

### Patient Characteristics

**Figure 1.**
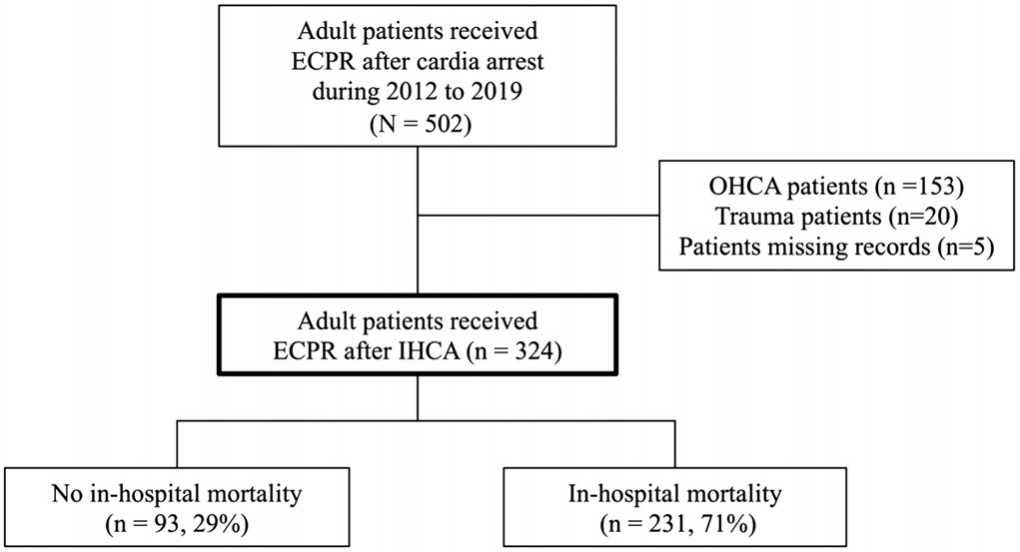
Study enrollment flowchart.

During the study period (January 2012–December 2019), 324 eligible patients who received ECPR after IHCA were enrolled in this study, and among them 231 (71%) died before hospital discharge (Figure 1). Table 1 presents the baseline characteristics of the participants. In the overall cohort, 121 patients (37.3%) had an initial shockable rhythm, and 265 patients (82%) were presumed to have a medical illness. Patients who survived to discharge after receiving ECPR for IHCA, when compared with the non-survivors, had a higher frequency of hypertension (62/93 [66.7%] vs 121/231 [52.3%], *P* = 0.03), presented more frequently with an initial shockable rhythm (50/93 [53.8%] vs 71/231 [30.7%], *P* < 0.001), had a shorter low flow duration (28 minutes vs 38 minutes, *P* < 0.001), and the cardiac arrest was more frequently presumed to be of a medical cardiogenic or surgical cardiogenic origin (62/93 [66.7%] vs 125/231 [54.1%]; 7/93 [7.5%] vs 16/231 [6.9%], *P* = 0.03). The duration of ECMO support was shorter (2 days vs 4 days, *P* = .002), and the total hospital length of stay was longer (15 days vs 3 days, *P* < .001) in the survival group. No significant intergroup differences between survivors and non-survivors were detected in terms of age, gender, body weight, BMI, history of comorbidities besides hypertension, witnessed arrest, or time of day.

**Table 1.** Comparison of basic characteristics of in-hospital cardiac arrest patients receiving extracorporeal cardiopulmonary resuscitation with or without survival to discharge.

	Patients died before discharge (n = 231)	Patients survived to discharge (n = 93)	*P*
	N (%) / median (IQR)	N (%) / median (IQR)	
Male	158 (69.6)	69 (30.4)	0.35
Age (year)	63.20 (50.40–70.00)	59.90 (50.35–66.85)	0.17
BW (kg)	67.40 (24.92–27.49)	65.00 (59.55–75.55)	0.88
BMI (kg/m^2^)	24.92 (21.98–27.49)	24.28 (22.44–26.70)	0.53
Past comorbidities			
HTN	121 (66.1)	62 (33.9)	0.03
DM	91 (67.9)	43 (32.1)	0.27
COPD	6 (85.7)	1 (14.3)	0.68
CAD	79 (66.9)	39 (33.1)	0.20
CHF	50 (75.8)	16 (24.2)	0.46
Renal insufficiency	50 (79.4)	13 (20.6)	0.12
CVA	13 (81.2)	3 (18.8)	0.57
Cancer	25 (86.2)	4 (13.8)	0.08
CPR			
Witnessed arrest	213 (70.8)	88 (29.2)	0.63
Initial shockable rhythm	71 (58.7)	50 (41.3)	<0.001
Time of day			
07:00–14:59	99 (66.9)	49 (33.1)	0.27
15:00–10:59	81 (74.3)	28 (25.7)	
23:00–06:59	51 (76.1)	16 (23.9)	
Low-flow duration (min)	38 (26–51)	28 (20.5–39)	<0.001
Presumed disease category			
Medical noncardiogenic	57 (73.1)	21 (26.9)	0.03
Medical cardiogenic	125 (66.8)	62 (33.2)	
Surgical cardiogenic	16 (69.6)	7 (30.4)	
Surgical noncardiogenic	33 (91.7)	3 (8.3)	
Duration of ECMO support (day)	4 (3–6)	2 (1–5)	0.002
Hospital length of stay (day)	3 (1–14)	15 (8–27)	<0.001

Dichotomous variables were reported as number (percentage) while continuous variables were reported as median (interquartile range).

*BMI*, body mass index; *BW*, body weight; *CAD*, coronary artery disease; *CHF*, congestive heart failure; *CPR*, cardiopulmonary resuscitation; *CVA*, cerebrovascular event; *DM*, diabetes mellitus; *ECMO*, extracorporeal membrane oxygenation; *ECPR*, extracorporeal cardiopulmonary resuscitation; *IHCA*, in-hospital cardiac arrest; *IQR*, interquartile range; *kg*, kilograms.

### External Validation of the RESCUE-IHCA Score

**Figure 2.**
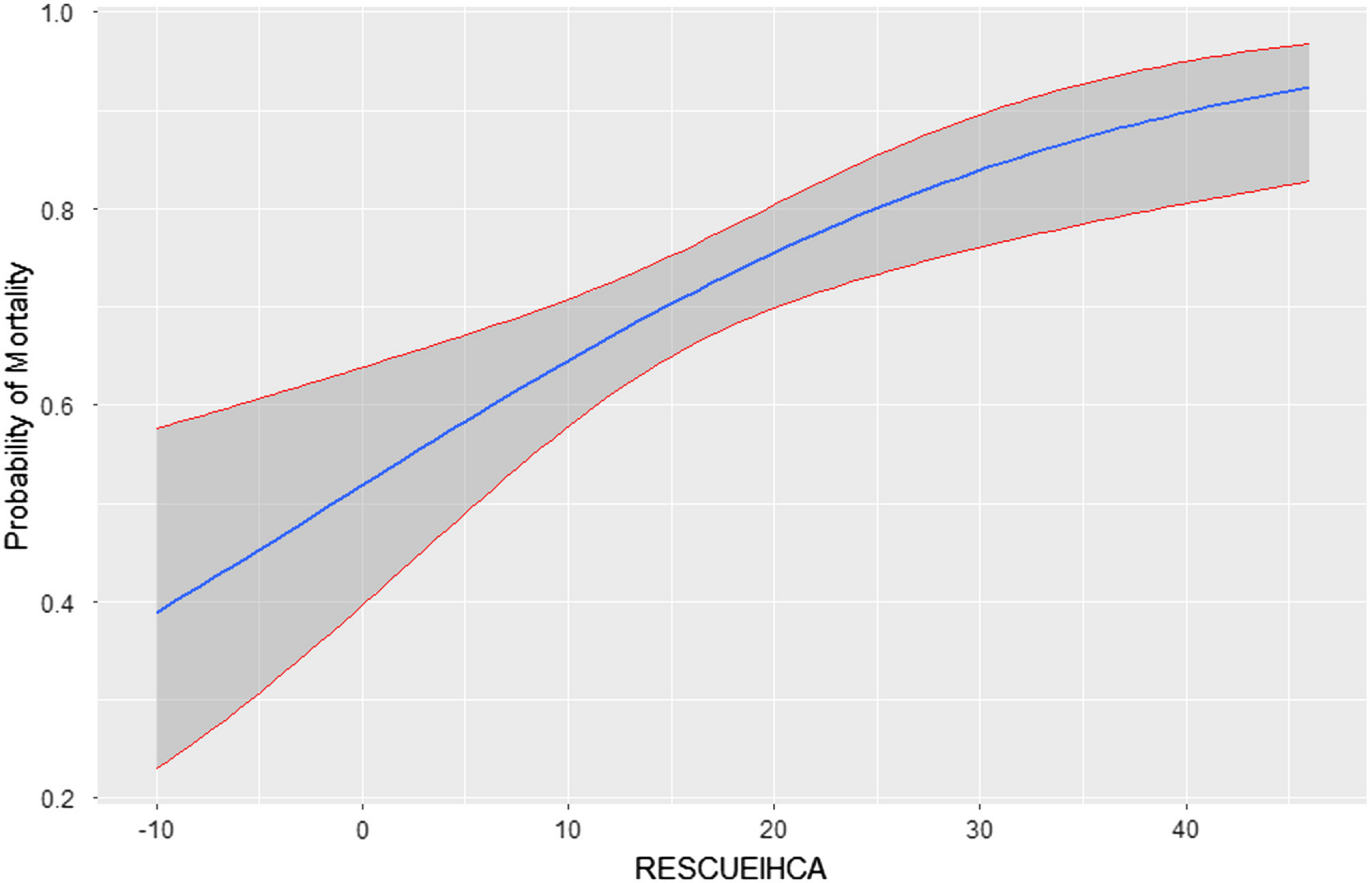
Predicted probability of death across RESCUE-IHCA score.

**Figure 3.**
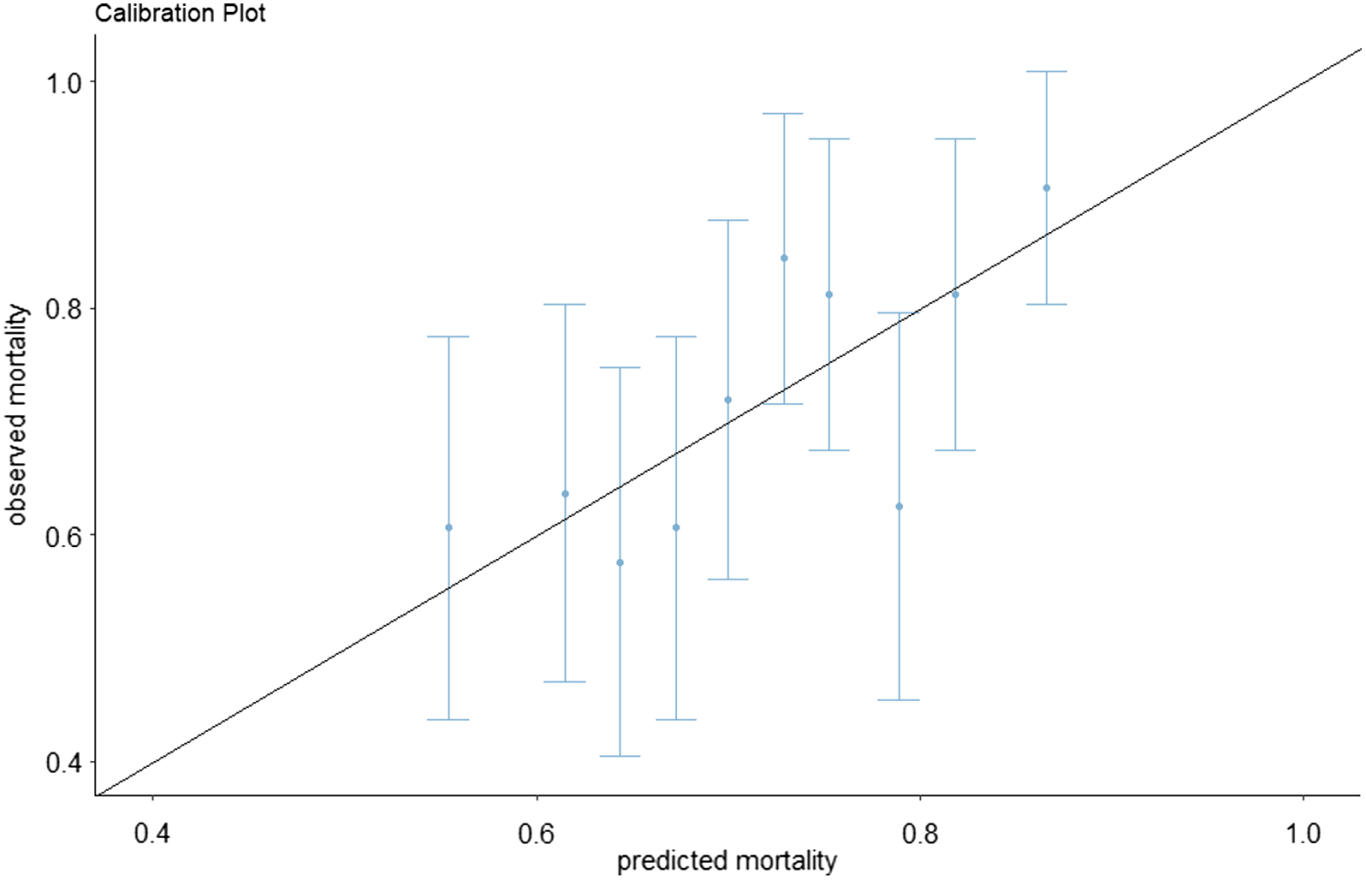
Calibration plot of observed vs predicted mortality from validated dataset.

The RESCUE-IHCA predictive model was externally validated among the 324 participants. The model discrimination was poor to acceptable (area under the curve 0.63 [95% CI 0.56–0.70]). The predicted probability of mortality ranged from 38–93% according to the RESCUE-ICHA score (Figure 2). Model calibration indicated good fit with the Hosmer-Lemeshow goodness-of-fit test (*P* = 0.91). The observed mortality in the study cohort vs the predicted mortality calculated from the RESCUE-IHCA score is presented in Figure 3. Other bin sizes were likewise tested without further improvement in fit.

### Significant Factors Associated with In-Hospital Death in the Current Cohort

To assess potential predictive factors in our cohort and compare them with the original RESCUE-IHCA score, we conducted univariate logistic regression for all variables, followed by multivariate regression for those variables with *P* < 0.1 (Table 2). The result showed that the mortality risk was positively associated with longer low-flow duration (odds ratio [OR] 1.02, 95% CI 1.01–1.03, *P* = .006), and negatively associated with an initial cardiac rhythm of VT (OR 0.14, 95% CI 0.04–0.51, *P* = .003), VF (OR 0.11, 95% CI 0.03–0.46, *P* = .003), or palpable pulse (OR 0.26, 95% CI 0.07–0.92, *P* = 0.04). Patient’s age, pre-existing renal insufficiency, timing of resuscitation, and disease category did not show significant associations with mortality. The Hosmer-Lemeshow test results showed a good fit (*P* = 0.66).

**Table 2.** Logistic regression model of risk factors associated with in-hospital death.

	N (%) / median (IQR)	Crude OR (95% CI)	*P*	Adjusted OR (95% CI)	*P*
Male	227 (70.1)	0.75 (0.44–1.29)	0.30		
Age (year)	59.7 (45.7–73.7)	1.01 (0.99–1.03)	0.28		
BW (kg)	66.55 (57.60–76.45)	1.00 (0.99–1.02)	0.94		
BMI (kg/m2)	24.64 (22.20–27.36)	1.01 (0.96–1.06)	0.80		
Past comorbidities					
HTN	183 (56.5)	0.55 (0.33–0.91)	0.02	0.70 (0.41–1.20)	0.19
DM	134 (41.4)	0.76 (0.47–1.23)	0.26		
COPD	7 (2.2)	2.45 (0.29–20.66)	0.41		
CAD	118 (36.4)	0.72 (0.44–1.18)	0.19		
CHF	66 (20.4)	1.23 (0.76–1.99)	0.41		
Renal insufficiency	63 (19.4)	1.70 (0.88–3.30)	0.12		
CVA	16 (4.9)	1.79 (0.50–6.43)	0.37		
Cancer	29 (9)	2.70 (0.91–7.99)	0.07		
CPR					
Witnessed arrest	301 (92.9)	0.67 (0.24–1.87)	0.45		
Presenting rhythm					
Asystole	42 (13)	reference		reference	
PEA	19 (5.9)	0.29 (0.06–1.45)	0.13	0.32 (0.06–1.70)	0.18
VT	83 (25.6)	0.11 (0.03–0.37)	<0.001	0.14 (0.04–0.51)	.003
VF	34 (10.5)	0.10 (0.03–0.38)	0.001	0.11 (0.03–0.46)	.003
Pulse (+)	146 (45)	0.24 (0.07–0.81)	0.02	0.26 (0.07–0.92)	0.04
Time of day					
07:00–14:59	148 (45.7)	reference			
15:00–10:59	109 (33.6)	0.69 (0.37–1.29)	0.24		
23:00–06:59	67 (20.7)	1.19 (0.60–2.35)	0.63		
Low flow duration (min)	37.6 (17.0–58.3)	1.03 (1.01–1.04)	0.001	1.02 (1.01–1.03)	.006
Presumed disease category					
Medical noncardiac	78 (24.1)	reference		reference	
Medical cardiac	187 (57.7)	0.74 (0.41–1.33)	0.32	1.23 (0.63–2.38)	0.55
Surgical cardiac	23 (7.1)	0.84 (0.30–2.33)	0.74	1.28 (0.43–3.75)	0.66
Surgical noncardiac	36 (11.1)	4.05 (1.12–14.63)	0.03	4.39 (1.17–16.46)	0.03

Dichotomous variables were reported as number (percentage) while continuous variables were reported as median (interquartile range). Variables with P < 0.1 in univariable logistic regression were adjusted.

*BMI*, body mass index; *BW*, body weight; *CAD*, coronary artery disease; *CHF*, congestive heart failure; *CPR*, cardiopulmonary resuscitation; *CVA*, cerebrovascular event; *DM*, diabetes mellitus; *IQR*, interquartile range; *kg*, kilograms; *N*, case number; *OR*, odds ratio; *PEA*, pulseless electrical activity; *VF*, ventricular fibrillation; *VT*, ventricular tachycardia.

## DISCUSSION

### Validation of the RESCUE-ICHA Score

In the present study, we performed temporal and geographical external validation of the RESCUE-IHCA scoring system in an Asian medical center equipped with standardized protocols for ECMO initiation. The performance of model discrimination (AUC 0.63) modestly decreased as compared with the original derivation and validation cohorts (AUC 0.72 [95% CI 0.68–0.76] and 0.68 [95% CI 0.61–0.75], respectively). The model’s performance may be attributed to the lack of significance of certain variables, including age, timing of resuscitation, the presumed disease category, and pre-existing renal insufficiency. We found that the low flow duration and the initial cardiac rhythm serve as significant predictors for the outcome, consistent with findings from previous observational studies and meta-analyses.^5^ ^,^ ^9^ ^–^ ^12^ ^,^ ^19^ Despite the single-center focus, the hospital is globally recognized as the second-largest facility for ECPR procedures, managing hundreds of cases each year. This study provides novel insights within a unique ethnic context.

### Individual Predictors of In-Hospital Death

Age was not a significant predictor in our study, possibly attributable to the small sample size. When comparing patient characteristics between studies, we observed a similar age distribution among non-survivors and survivors in the two cohorts (study cohort: 63.2 vs 59.9; RESCUE-IHCA cohort: 61 vs 58).

In contrast to the notable finding from the RESCUE-IHCA study, the influence of time of day on survival no longer persisted. During late night and early morning from 11 pm – 5 am, fewer survivors were observed in the RESCUE-IHCA cohort compared to the current cohort (study cohort: 16/93 [17.2%]; RESCUE-IHCA cohort: 27/306 [8.8%]). The current study was conducted at a tertiary medical center where ECPR initiation was protocolized regardless of timing. When a cardiac arrest occurs, a standardized hospital-wide emergency call activates a rapid response team of medical cardiologists, surgeons, and emergency physicians who promptly determine the need for ECPR.

A comprehensive discussion on ECPR implementation was provided by a prospective observational study conducted in Taiwan.^7^ Experienced surgeons and team members subsequently establish ECMO cannulation at the bedside if indicated. Despite reduced ward staffing during the night, survival rates were not significantly affected. A prior study conducted at a medical center in Taiwan revealed that the time of day had no impact on the survival outcome following an in-hospital cardiac arrest.^13^ Our study suggests that an experienced healthcare system with trained crew members operating in an established system can effectively mitigate the increased workload from decreased staffing during off-hours. Further studies are warranted to determine the impact of hospital caseload and experience while focusing on the outcomes of cardiac arrest patients who receive ECPR.

The RESCUE-IHCA study found that surgical cardiac, surgical non-cardiac, and medical-cardiac diseases were predictive factors for survival. However, the results of the current study only observed a relationship between surgical non-cardiac disease and in-hospital mortality, although this association did not reach statistical significance (Table 1). The surgical non-cardiac diseases in our study included aortic dissection, hypovolemia or hemorrhage, and intracranial hemorrhage, which may potentially derive less benefit from ECPR (Supplementary Table 1). The 2010 RESCUE-IHCA study included a higher proportion of surgical patients (610/1,075 [56.7%]), whereas the current study comprised a lower percentage of patients with surgical illnesses (59/324 [18.2%]). The disease category in the original RESCUE-IHCA study was automatically retrieved from Current Procedure Terminology (CPT) and International Classification of Diseases (ICD) codes, whereas in this study we manually reviewed the health charts to assess the ultimate etiology of the cardiac arrest.

Previous studies have identified “cardiac origin” or “presumed reversible cardiogenic etiology” as critical selection criteria for IHCA patients receiving ECPR, without specifying the diagnostic process or disease-categorization method.^7^ ^–^ ^9^ ^,^ ^15^ ^,^ ^16^ ^–^ ^18^ These divergent findings highlight the complexity of the presumed etiology of arrest. Concerns persist regarding the potential misinterpretation of diagnoses, which could result in the inappropriate initiation of ECPR in acute scenarios. Establishing and validating a standardized disease-categorization system for IHCA patients receiving ECPR is a crucial challenge that may significantly improve outcomes in the future.

Renal insufficiency was not identified as a significant predictor for mortality in this study. Our study included fewer patients with pre-existing renal insufficiency in both the mortality and survival groups (non-survivors vs survivors in the study cohort: 50/231 [21.6%] vs 13/93 [14.0%]; RESCUE-IHCA cohort: 193/769 [25.1%] vs 49/306 [16.0%]). Previous studies conducted in an East Asian population exhibited similar proportion of patients with renal insufficiency to our study.^20^ ^,^ ^21^ The proportion of patients with renal disease influences the results; however, renal function still plays a crucial role in prognostication.

#### Application of the RESCUE-IHCA score in real-world scenarios

The overall survival rate of IHCA patients treated with ECPR was 28.7% in the study, which approximates to real-world conditions according to recent observational studies and systemic reviews wherein reported survival rates ranged from 23.1–40%.^22^ ^–^ ^25^ In conclusion, the performance of the RESCUE-IHCA score was modestly compromised in this single-center cohort. Although a suboptimal validation of RESCUE-IHCA score in this cohort might not indicate its prognostic performance in other populations with different characteristics, suggestions were made for personalized decisions considering the patient’s arrest etiology, clinical status, and the institutional capacity and experiences.

## LIMITATIONS

There were several limitations to this study. Firstly, missing data is a common issue in retrospectively collected variables, and this study was no exception. Patients with missing outcomes were excluded from the beginning, compromising the size of the cohort. Secondly, the small sample size may have led to higher variability, which might not accurately reflect the real-world situation. Thirdly, neurological outcomes were not assessed due to a considerable amount of missing data. Further large-scale validation studies should be performed to conducted to assess the universal applicability of this score.

## CONCLUSION

In the current study cohort, the RESCUE-IHCA score did not predict outcomes better than the originally validated cohort, with low flow duration and initial rhythms persisting as consistent predictive factors. The method of disease categorization in IHCA patients and the differences in hospital experience may have influenced these outcomes. Although the six-factor score carried some advantages, significant limitations were present. Further research is needed to explore the impact of hospital experience and standardized diagnostic criteria for cardiac origin IHCA on the ECPR outcomes.

## Supplementary Information





## Notes

**Ethics committee approval**: This research is approved by the Institutional Review Board (202306052RIND). The requirement for informed consent was waived because of the retrospective nature.

## Notes

**Availability of data and materials**: The datasets used or analyzed during the current study are available from the corresponding author on reasonable request.
