# Supplementary material for: External Validation of the RESCUE-IHCA Score as a Predictor for In-Hospital Cardiac Arrest Patients Receiving Extracorporeal Cardiopulmonary Resuscitation
Source: West J Emerg Med. 2024 Oct 4;25(6):894–902. doi: 10.5811/westjem.18601 (PMC11610726; doi:10.5811/westjem.18601)
Supplement: Supplementary file 1 [file wjem-25-894-s001.docx]

| **Supplemental Table 1.** Types of cardiogenic and non-cardiogenic causes of IHCA | | |
| --- | --- | --- |
|  | N | % |
| Cardiogenic cause | 210 | 64.8 |
| Medical cause | 187 | 57.7 |
| Acute coronary syndrome | 110 | 34.0 |
| Myocarditis | 7 | 2.2 |
| Cardiomyopathy | 35 | 10.8 |
| Right ventricular failure | 11 | 3.4 |
| Arrythmia | 9 | 2.8 |
| Valvular disease | 15 | 4.6 |
| Surgical cause | 23 | 7 |
| Cardiac rupture with tamponade | 8 | 2.5 |
| Post-cardiac surgery | 14 | 4.3 |
| Post-heart transplant | 1 | 0.3 |
| Non-cardiogenic cause | 114 | 35.1 |
| Medical cause | 78 | 24.0 |
| Transplant rejection | 6 | 1.9 |
| Pulmonary embolism | 17 | 5.2 |
| Amniotic fluid embolism | 1 | 0.3 |
| Infection | 28 | 8.6 |
| Thyroid storm | 2 | 0.6 |
| Airway obstruction | 10 | 3.1 |
| Acute respiratory distress syndrome | 5 | 1.5 |
| Electrolyte disturbances | 5 | 1.5 |
| Intoxication | 8 | 2.5 |
| Surgical cause | 36 | 11.1 |
| Aortic dissection | 19 | 5.9 |
| Hypovolemia (Bleeding) | 16 | 4.9 |
| Intracranial hemorrhage | 1 | 0.3 |
| N=case number, IHCA=in-hospital cardiac arrest |  |  |
